# Riddle Me This: Acalculous Cholecystitis as an Unusual Complication of Immunoglobulin M Negative Mononucleosis

**DOI:** 10.7759/cureus.2505

**Published:** 2018-04-19

**Authors:** Philipp Höhn, Chris Braumann, Waldemar Uhl, Andreas M Luu

**Affiliations:** 1 Department of General and Visceral Surgery, Katholisches Klinikum Bochum - St. Josef Hospital, Ruhr University Bochum

**Keywords:** infectious mononucleosis, splenectomy, acalculous cholecystitis, epstein-barr virus (ebv)

## Abstract

Infectious mononucleosis is a common disease of the adolescent caused by the Epstein-Barr virus (EBV). We present a rare case of a male adult with acalculous cholecystitis due to infectious mononucleosis. A correct diagnosis was challenging due to a false negative antibody test. Laboratory values were significant for a marked lymphocytosis and an early Immunoglobulin G (IgG) response without initial Immunoglobulin M (IgM) elevation. However, IgM antibodies were elevated two weeks later. Symptoms resolved quickly under symptomatic therapy. Antibody level patterns in asplenic patients with infectious mononucleosis are characterized by an atypical course with a delayed rise in IgM antibodies, which complicates the correct diagnosis of an EBV-induced acalculous cholecystitis.

## Introduction

Infectious mononucleosis is a common disease of the adolescent caused by the Epstein-Barr virus (EBV), a widely disseminated herpes virus (Type IV). Frequent symptoms are fatigue and dysphagia while clinical signs include fever, adenopathy, pharyngitis, and atypical lymphocytosis [1]. Diagnosis is usually established by clinical signs and supported by antibody detection. The latter can be misleading in immunocompromised patients. We present the rare case of an adolescent patient with acalculous cholecystitis due to infectious mononucleosis. The correct diagnosis was challenging due to a negative antibody test after a splenectomy he had undergone years ago.

## Case presentation

**Figure 1:**
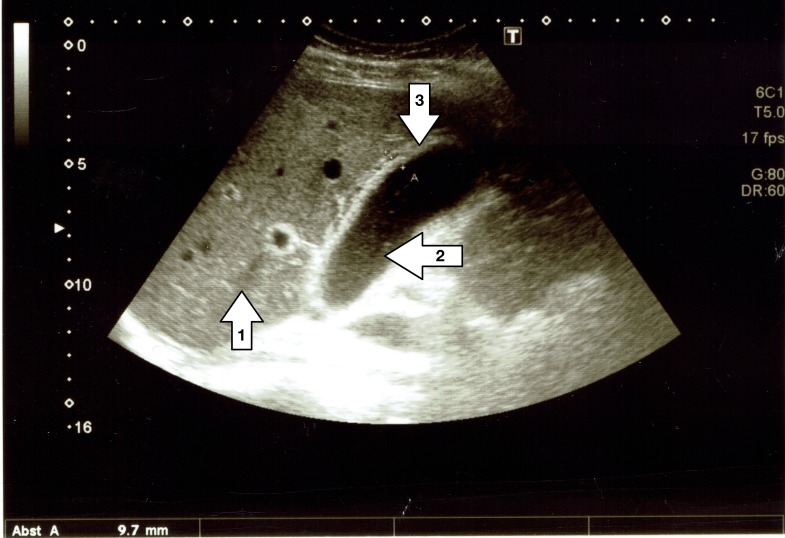
Ultrasound image of patient's gallbladder The figure shows the patient's liver (arrow 1) and gallbladder (arrow 2) on admission day. While no stones can be found inside, the gallbladder wall (arrow 3) presents as multilayered and thickened (9.7 mm).

A 24-year-old Caucasian male presented to our outpatient clinic with fever and pain in the right upper abdominal quadrant. He had a history of a recent upper respiratory tract infection, which was treated with oral amoxicillin. The patient also had a history of left adrenal gland resection, distal pancreatectomy, and splenectomy due to a large pheochromocytoma two years earlier. His past medical history was otherwise unremarkable. A clinical examination revealed a tenderness in the epigastric abdomen and a cervical lymphadenopathy. An ultrasound examination revealed a thickened gallbladder wall as a sign of acute cholecystitis without evidence of gallstones or sludge, as shown in Figure 1.

**Figure 2:**
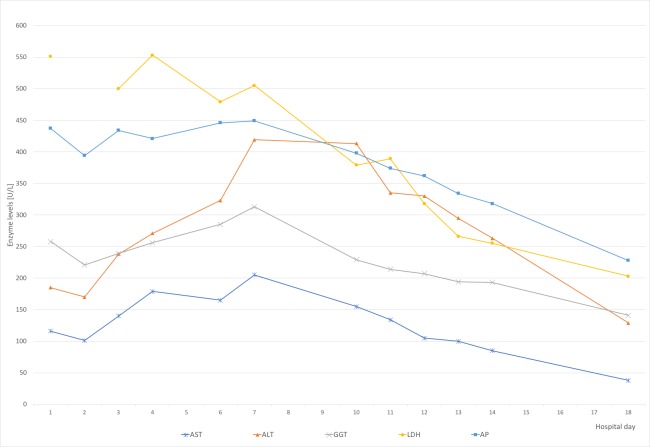
Course of liver enzymes AST: aspartate aminotransferase, ALT: alanine aminotransferase, GGT: gamma-glutamyltransferase, LDH: lactate dehydrogenase, AP: alkaline phosphatase

Due to increasing leukocytosis, abdominal pain, and the history of a huge pheochromocytoma, computed tomography (CT) was performed. This confirmed an acalculous cholecystitis and showed a generalized lymphadenopathy. Laboratory values were significant for: white blood cell (WBC) count 23.940/µl (range: 4.500-9.500/µl), thrombocytes 426.000/µl (150.000-400.000), lactate dehydrogenase (LDH) 438 U/l (135-225), aspartate aminotransferase (AST) 116 U/l (10-50), alanine aminotransferase (ALT) 185 U/l (10-50), gamma-glutamyltransferase (GGT) 258 U/l (10-71), alkaline phosphatase (AP) 437 U/l (40-129), c reactive protein (CRP) 10.2 mg/l (< 5mg/l). Table 1 summarizes the laboratory values throughout the hospital stay. Figure 2 presents the course of the patients' liver enzymes.

**Table 1:** Overview of liver enzymes Table showing the WBC and liver enzyme levels on admission and discharge. Pathological values on admission and discharge are marked in bold. WBC: white blood cell count, LDH: lactate dehydrogenase, AST: aspartate aminotransferase, ALT: alanine aminotransferase, GGT: gamma-glutamyltransferase, AP: alkaline phosphatase

	AST (U/l)	ALT (U/l)	GGT (U/l)	AP (U/l)	LDH (U/l)	WBC (/µl)
Admission day	116	185	258	437	551	27260
Max. value	205	419	313	449	553	34920
Discharge day	38	129	141	228	203	11770
Range	10-50	10-50	10-71	40-129	135-225	4600-9500

The mononucleosis enzyme-linked immunosorbent assay (ELISA) showed elevated virus capsid antigen (VCA) immunoglobulin G (IgG) levels (39 U/l; range < 0 U/l) and normal VCA immunoglobulin M (IgM) levels. The heterophile antibody test for mononucleosis IgM antibodies was positive. We performed a cytomegalovirus (CMV) ELISA, which showed normal values for IgG and IgM. Epstein-Barr virus (EBV)-specific nuclear antigen (EBNA) antibodies were negative. A VCA IgM control test two weeks later showed elevated VCA IgM antibodies. The peripheral blood smear confirmed atypical lymphocytosis. Under symptomatic therapy, the patient’s state improved rapidly and he was discharged on the 17^th^ day after admission in good general condition.

## Discussion

Infectious mononucleosis is a common disease of the adolescent caused by EBV, a widely disseminated herpes virus type IV. About 90% to 95% of adults worldwide are EBV positive. The virus is spread by intimate contact between persons; hence, the common name “kissing disease.” Less than 10% develop a clinical infection. The traditionally reported peak incidence of clinical infections lies between the age of 15 and 24 years. Most adults are immune to the infection due to prior exposure to EBV [2-4].

Our case was unique due to several unusual conditions. First, acalculous cholecystitis is an extremely rare complication of an EBV infection. Second, the correct diagnosis of acute mononucleosis as an underlying cause for cholecystitis was challenging due to alternated laboratory values. While the test for heterophile antibodies was positive and the patient was clearly presenting with clinical signs of infectious mononucleosis, the EBV VCA IgM was surprisingly negative at first.

Diagnosis is usually established by clinical signs and supported by the heterophile test. Further laboratory diagnostic studies include the detection of VCA IgG and IgM antibodies via ELISA. The detection of EBNA and early antigen (EA) via Immunoblot can also be performed. In case of VCA, IgM antibodies allow the diagnosis of an acute infection, as they are usually present at the onset of clinical symptoms. They last for about four months. IgG antibodies are usually also positive after a few days of clinical onset and persist lifelong as a marker of EBV infection. EBNA antibodies appear six to 12 weeks after the infection and persist lifelong. Their presence basically rules out an acute infection.

Han et al. described a total of three patients who developed post-splenectomy infectious mononucleosis caused by CMV. The patients had undergone splenectomy as a treatment of hereditary spherocytosis years earlier. Remarkably, the authors found a strong response of IgG antibodies against CMV in the early phase of the infection, followed by an increased level of IgM antibodies 11 weeks after the peak of IgG antibodies. This lead to an inverted course of antibody levels compared to immunocompetent patients. In contrast to other immunodeficient states, the infections resolved spontaneously without anti-CMV therapy [5-6].

Research shows that splenectomy leads to a diminished antibody response to bacterial polysaccharide vaccines. Data suggest that the IgM response is more impaired than the IgG response. Han et al. suspected a similar mechanism in case of viral infections. This is supported by findings that show that in human blood IgM memory B cells circulate splenic marginal zone B cells. Furthermore, asplenic humans have undetectable IgM memory B cells [7-9].

Our patient presented in a quite similar manner to the cases Han et al. described. We found a marked lymphocytosis and an early IgG response with no initial IgM elevation. Our patient also recovered without specific antiviral therapy. We interpret this as a hint that antibody level patterns in asplenic patients infected with EBV might be similar to those in CMV.

## Conclusions

This is the first reported case of infectious mononucleosis caused by EBV with acalculous cholecystitis in a patient with a history of splenectomy. Cholecystectomy is not indicated, as symptoms can be expected to resolve spontaneously. In patients with a history of splenectomy, VCA IgM antibodies can be negative in the acute phase and rise approximately two weeks after the onset of symptoms, whereas the IgG antibodies are elevated earlier. Infectious mononucleosis caused by either EBV or CMV in patients with a history of splenectomy is a rare disease. Knowledge of altered antibody patterns is crucial for a correct diagnosis.
